# Geometric Morphology of the Coracoacromial Ligament: A Cadaveric Study

**DOI:** 10.1155/2019/3024769

**Published:** 2019-02-10

**Authors:** Mehmet Demir, Bülent Güneri

**Affiliations:** ^1^Department of Anatomy, Faculty of Medicine, Kahramanmaras Sütcü Imam University, Kahramanmaras, Turkey; ^2^Department of Orthopedics and Traumatology, Faculty of Medicine, Kahramanmaras Sütcü Imam University, Kahramanmaras, Turkey

## Abstract

The coracoacromial ligament (CAL), which restrains superior displacement of humeral head, connects the acromion and coracoid process. Due to the ligament's variations and its role in shoulder pain, CAL was investigated in this study. Sixty shoulders of 34 cadavers, from persons aged 61-98 (80.95 ± 8.81) years at death time, were dissected. The lengths of lateral (LBL) and medial borders (MBL), widths of acromial (AIW) and coracoid insertions (CIW), and thicknesses of lateral (LSTAI) and medial (MSTAI) sides of acromial insertions were measured by digital caliper. The data were subjected to statistical analysis. 24 (40%) V-shaped, 12 (20%) broad-banded, 9 (15%) quadrangular, 9 (15%) Y-shaped, and 6 (10%) multiple-banded types were identified. The mean total LBL, MBL, AIW, CIW, LSTAI, and MSTAI were 34.94 ± 4.59 mm, 33.58 ± 5.31 mm, 29.82 ± 9.48 mm, 12.62 ± 3.95 mm, 1.29 ± 0.17 mm, and 0.90 ± 0.22 mm, respectively. The mean LBL (39.12 ± 4.29 mm), MBL (36.48 ± 3.9 mm), and CIW (37.01 ± 3.39 mm) were significantly greatest in quadrangular type (p<0.001). The mean AIW was slightly greatest in quadrangular type (p=0.069). The mean LSTAI was significantly greatest in multiple-banded type (1.45 ± 0.10 mm, p<0.001) whereas the mean MSTAI was significantly greatest in quadrangular type (1.23 ± 0.23 mm, p<0.001). CAL is quite variable regarding morphology, dimensions, and insertion features. Despite common knowledge, MSTAI and MBL of CAL can be greater than lateral counterparts in some types. To obtain complete release of CAL at acromion, the clearance of ligament fibers in an area with the dimensions of around 16 mm in mediolateral and 15 mm in anteroposterior direction, beginning from the lateral edge of acromial insertion, is recommended.

## 1. Introduction

The shoulder joint is a highly mobile and complicated joint connecting upper extremity and thorax [1]. Shoulder pain occurs in up to 67% of the population at least once in lifetime [2]. Its accurate diagnosis is difficult due to unique anatomy of the shoulder joint [1]. During regular glenohumeral joint motion, all rotator cuff muscles operate harmoniously and stabilize the humeral head to the center of glenoid fossa [3]. Rotator cuff dysfunction (i.e., weak dynamic stabilization of humeral head) and elevation of humeral head by deltoid muscle activity are suggested to cause subacromial impingement (SAI) which is a common musculoskeletal system problem and one of the most frequent causes of shoulder pain, significantly reducing the life quality and work capacity of individuals [4, 5]. Narrowing of the subacromial space due to various external causes (such as hooked acromion, os acromiale, osteophytes of acromioclavicular joint) leads to SAI, which can result in rotator cuff tendinitis and consequent cuff tear as well [3, 6].

The coracoacromial ligament (CAL), which restrains superior displacement of humeral head, is a component of coracoacromial arch, an osseoligamentous structure that extends between acromion and coracoid process of scapula [7–9]. Since its role in SAI has been defined, variations of CAL in terms of morphology, acromial and coracoid insertion features, and relationship with surrounding anatomical structures are of clinical interest but have been reported in few studies [7, 10–13]. Additionally, the reported prevalence of CAL types with regard to band number and shape is diverse [14]. With this respect, the present cadaveric study investigates the dimensions of CAL, variations in ligament morphology, and coracoid and acromial insertions which are crucial for insight into surgical management of subacromial impingement.

## 2. Material and Methods

Thirty-four cadavers, from persons aged 61-98 years (mean 80.95 ± 8.81 years) at the time of death, were dissected in this study which includes 60 shoulders (i.e., bilateral dissection in 26 and unilateral dissection in 8 cadavers). The dissected shoulders were free of structural deficiency, previous injury, or surgical intervention while eight shoulders were excluded due to structural deficiencies. The dissections were carried out in the Department of Anatomy at the University of Cologne, Germany. Informed consent was obtained prior to or at the time of body donation by the Anatomy Department.

Dissection of each shoulder commenced with removal of skin and subcutaneous fat using an incision, beginning from superior aspect of shoulder and extending distally on anterior aspect of shoulder throughout proximal one-third of upper arm. Afterwards, deltoid muscle and subacromial bursa were excised to expose CAL and its insertions. Each specimen was categorized into five anatomical variations according to the number of band(s), shape formed by the band(s), and acromial insertion of the ligament, as described by Kesmezacar et al. [12]: Y-shaped, broad-banded, quadrangular, V-shaped, and multiple-banded. Broad-banded type is characterized by approximately equal width of acromial and coracoid insertion. Quadrangular type includes a single band attaching to coracoid process with greater width compared to acromial insertion. Y-shaped type features single-banded insertion on acromion and two-banded insertion on coracoid process. V-shaped type includes two-banded, narrower insertion on acromion and two-banded, wider insertion on coracoid process. Multiple-banded type contains three or more bands.

The length of ligaments' lateral and medial borders from acromial insertion to coracoid insertion, the mediolateral dimensions (widths) of ligaments' acromial and coracoid insertions, and anteroposterior dimensions (thicknesses) of acromial insertion at the lateral and medial sides were measured by digital caliper to the nearest 0.01 mm.

All statistical analyses were conducted using IBM SPSS for Windows version 22 (IBM Corporation, Armonk, New York, United States). Descriptive statistics were used for the means and standard deviations. Shapiro-Wilk test was applied to evaluate data distribution. In the normally distributed data, ANOVA was used for the multiple comparisons. Post hoc comparisons were made using Tukey HSD test and Tamhane T2 test. For the comparison of two independent variables, independent samples t test was used. An exact test was applied to determine the relationships between the categorical variables. The critical significance was accepted as p<0.05.

## 3. Results

**Figure 1:**
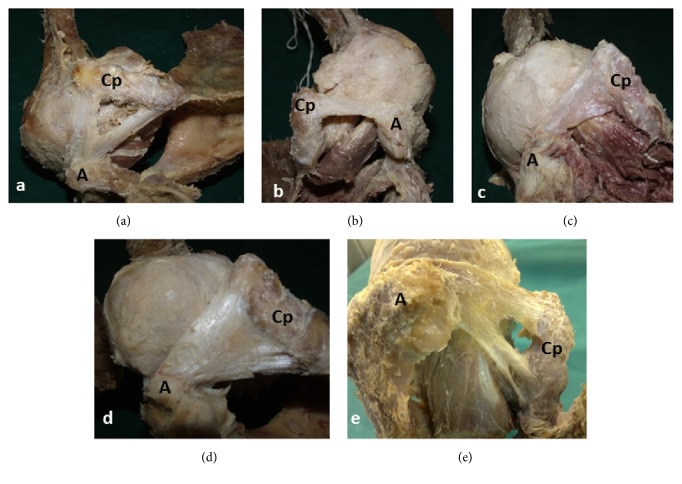
Photographs representing each variation from shoulders dissected in the present study (A: acromion; Cp: coracoid process): (a) Y-shaped, (b) broad-banded, (c) quadrangular, (d) V-shaped, (e) multiple-banded.

All types of CAL variations were observed in this study: Y-shaped type in 9 shoulders (15%; 3 from male, 6 from female cadavers) (Figure 1(a)), broad-banded type in 12 shoulders (20%; 3 from male, 9 from female cadavers) (Figure 1(b)), quadrangular type in 9 shoulders (15%; 6 from male, 3 from female cadavers) (Figure 1(c)), V-shaped type in 24 shoulders (40%; 12 from male, 12 from female cadavers) (Figure 1(d)), and multiple-banded type in 6 shoulders (10%; all from female cadavers) (Figure 1(e)). In 10 cadavers (38.46%) among 26 cadavers dissected bilaterally, the ligaments on both sides were nonidentical in terms of variation. Among 16 cadavers (61.54%) involving identical ligaments on both sides, there were 6 cadavers (37.5%) with V-shaped type, three cadavers (18.75%) with quadrangular type, 3 cadavers (18.75%) with broad-banded type, 2 cadavers (12.5%) with Y-shaped type, and 2 cadavers (12.5%) with multiple-banded type CAL.

The mean values of lateral border length (LBL), medial border length (MBL), coracoid insertion width (CIW), and acromial insertion width (AIW) varied among ligament types (Table 1). The mean LBL, MBL, and CIW measurements were significantly greatest in quadrangular type. The mean AIW was greater in quadrangular type compared to other types; however, the difference was not significant. The mean values of Y-shaped and broad-banded types were below the total mean value (TMV) regarding LBL, MBL, CIW, and AIW. The mean lengths of lateral borders in V-shaped and multiple-banded types were below TMV while the measurements of other three parameters were above TMV.

The mean values of lateral side thickness of acromial insertion (LSTAI) were significantly above TMV in multiple-banded, Y-shaped, and broad-banded types whereas the mean value of medial side thickness of acromial insertion (MSTAI) was significantly greater than TMV in quadrangular type (Table 2). On the other hand, measurements in quadrangular and V-shaped types demonstrated significantly low mean LSTAI compared to TMV. Regarding mean MSTAI, V-shaped and multiple-banded types approximated TMV while measurements in broad-banded and Y-shaped types were significantly less than the TMV.

The ratio of LBL to MBL, CIW to AIW, and LSTAI to MSTAI were calculated in this study as well (Table 3). LBL/MBL ratio was significantly greatest in broad-banded type which was followed by Y-shaped, quadrangular, V-shaped, and multiple-banded types in descending order. The latter two variations were below TMV. The ratio of CIW to AIW was significantly highest in V-shaped type. The ratios were above TMV in multiple-banded, quadrangular, and Y-shaped types while they were below TMV in broad-banded type. The ratio of LSTAI to MSTAI was significantly greatest in Y-shaped type which was followed by broad-banded and multiple-banded types having mean values above TMV.

## 4. Discussion

CAL acts as a static restraint against superior translation of humeral head in association with acromion and coracoid process, forming the coracoacromial arch [7, 15]. Since Neer defined CAL as a pain source in SAI, the anatomical features of the ligament and the ligament's insertions to acromion and coracoid process called more attention [3]. Although the release of CAL has been commonly performed in acromioplasty, this intervention is controversial since the humeral head is prone to displace superiorly following release of the ligament [3, 16].

In many studies, significant variations of CAL were defined with regard to the course of its fibers (Table 4). The current study included approximately 10% multiple-banded ligament, which was the least encountered type. This result is compatible with the results reported by Kesmezacar et al. [12], Holt and Allibone [10], but contrary to the results of Alraddadi et al. [13]. The current study differs from the other studies in the literature regarding the most common type, V-shaped CAL. We suggest that the discrepancy is attributable to similar forms of two-banded (V-shaped and Y-shaped) variations which might be interpreted differently by examiners, i.e., interobserver variability. Postnatal change in ligament morphology due to developmental factors, as described by Kopuz et al. [17], is another possible cause to be mentioned for the explanation of the discrepancy between studies since these factors may vary between human populations.

As the distinction of V-shaped and Y-shaped types is observer-dependent and V-shaped ligament is a more recently described variation [12], the comparison of two-banded types' prevalence in the current study with the prevalence reported in earlier studies can be practical. Two-banded variations constitute 55% of dissected shoulders in the current study. This result is consistent with the review reported on CAL which mentioned that two-banded type had been encountered in 42 to 75% of shoulders [14].

The presence of identical CAL types on both sides was common in the current study since it was detected in more than 60% of the cadavers dissected for bilateral CAL inspection. Five variations were identified while V-shaped type was the common variation among cadavers with bilateral identical CAL types. Kesmezacar et al. [12] reported a similar rate, 64%, regarding identicalness of ligaments types in both shoulders.

The measurements of the previous studies and current study consistently indicated that lateral border of CAL was longer than the medial border considering TMV [13, 18]. Unlike the cadaveric study of Kesmezacar et al. [12] which reported the greatest mean LBL in broad-banded type, mean LBL as well as mean MBL was highest in quadrangular type in the current study. This study distinctly compares the mean LBL and MBL regarding CAL morphology and reports that mean MBL was slightly higher than mean LBL in V-shaped and multiple-banded types.

Different mean values of CIW disregarding CAL variations have been mentioned in a number of researches [13, 18]. On the other hand, Holt and Allibone [10] reported the mean CIW with regard to CAL types. Multiple-banded type, represented by one shoulder in the study, had the widest attachment (44 mm). The mean values of CIW were reported for quadrangular, Y-shaped, and broad-banded types as 32.8 ± 4.6 mm, 31.2 ± 4.5 mm, and 20.0 ± 3.4 mm, respectively. Unlike the latter study, the current study included 10% multiple-banded type which owned the second highest mean CIW while quadrangular type had the greatest insertion width. Broad-banded type featured the lowest mean CIW in both studies.

Several studies on CAL reported various mean values of AIW [7, 13, 18]. Holt and Allibone [10] mentioned 21 mm AIW for multiple-banded type, but 19.4 ± 4.5 mm, 19.4 ± 4.3 mm, and 19.0 ± 3.9 mm mean values of AIW for Y-shaped, quadrangular, and broad-banded types, respectively. Kesmezacar et al. [12] indicated that the mean values of AIW determined in their study were 15.60 ± 2.7 mm in Y-shaped, 15.44 ± 2.8 mm in multiple-banded, 14.18 ± 4.7 mm in quadrangular, 13.66 ± 3.5 mm in broad-banded, and 11.88 ± 2.4 mm in V-shaped types. Similar to the results of the latter two studies, the mean AIW was lowest in broad-banded group. It was highest in quadrangular type in the current study, unlike the aforementioned studies. The mean CIW to AIW ratio ranged between 1.42 ± 0.50 (the mean value of broad-banded type) and 2.92 ± 1.06 (the mean value of V-shaped type). These findings support the conventional knowledge that coracoid insertion is significantly wider than acromial insertion in all CAL variations.

Incomplete resection of CAL during subacromial decompression surgery may cause unfavorable outcome; therefore, meticulous identification of CAL is crucial, particularly in shoulders with more than one band and operated using arthroscope [7, 19]. Besides, the comprehension of morphologic dimensions of acromial insertion expectedly facilitates its release. According to the measurements of the current study, we recommend the clearance of CAL fibers in an area with the dimensions of around 16 mm in mediolateral and 15 mm in anteroposterior direction, beginning from the lateral edge of acromial insertion which is quite accessible during surgical procedures, in order to make sure that complete CAL release is achieved at acromial insertion of CAL.

Since a part of SAI occurrence is attributed to lateral bands' proximity to rotator cuffs and relatively thicker structure, the thickness on lateral aspect of acromial insertion seems to have clinical significance [7, 12]. With this respect, the mean lateral side thickness was compared with the mean medial side thickness of acromial insertions, regarding CAL variations in the current study. The lateral sides were significantly thicker than the medial sides of acromial insertion in total. This was also valid for four variations of CAL other than quadrangular type which demonstrated slightly thicker medial acromial insertion on medial side compared to lateral. Although the condition of rotator cuffs has not been evaluated in the current study, the latter finding of this study can be supported by nonexistence of cuff pathology in quadrangular type in the cadaveric study of Kesmezacar et al. [12].

## 5. Conclusion

CAL is a quite variable anatomical structure in terms of morphology, dimensions, and insertion features. Despite common opinion and knowledge, medial side thickness of acromial insertion and medial border length of CAL can be greater than lateral counterparts in some ligament types. To obtain complete release of CAL at acromion, the clearance of ligament fibers in an area with the dimensions of around 16 mm in mediolateral and 15 mm in anteroposterior direction, beginning from the lateral edge of acromial insertion, is recommended.

## Figures and Tables

**Table 1:** The mean lengths (mm) of medial and lateral borders and the mean widths of coracoid and acromial insertions according to CAL types.

	Y-shaped	Broad-banded	Quadrangular	V-shaped	Multiple-banded	Total	p
	n=9	n=12	n=9	n=24	n=6	n=60	
LBL	34.30 ± 4.35	34.52 ± 5.13	39.12 ± 4.29^e^	34.50 ± 4.12	32.30 ± 3.32^c^	34.94 ± 4.59	**0.035** **∗**
MBL	32.16 ± 4.27	27.35 ± 5.74^c,d,e^	36.48 ± 3.90^b^	35.50 ± 3.62^b^	34.13 ± 4.12^b^	33.38 ± 5.31	**p<0.001** **∗**
CIW	26.82 ± 2.56^b,c,d,e^	13.99 ± 3.43^a,c,d,e^	37.01 ± 3.39^a,b^	34.96 ± 5.22^a,b^	34.68 ± 3.70^a,b^	29.82 ± 9.48	**p<0.001** **∗**
AIW	10.82 ± 1.75	10.51 ± 2.80^c^	14.36 ± 0.89	13.45 ± 4.93	13.63 ± 4.71	12.62 ± 3.95	0.069

LBL: lateral border length; MBL: medial border length; CIW: coracoid insertion width; AIW: acromial insertion width.

Data are reported as mean ± SD. ANOVA; *α*: 0.05; independent t test; *∗*difference is statistically significant; post hoc; Tukey HSD test, Tamhane T2 test.

^a^ Difference with Y-shaped is statistically significant.

^b^ Difference with broad-banded is statistically significant.

^c^ Difference with quadrangular is statistically significant.

^d^ Difference with V-shaped is statistically significant.

^e^ Difference with multiple-banded is statistically significant.

**Table 2:** The mean thickness measurements (mm) of acromial insertions according to CAL types.

	Y-shaped	Broad-banded	Quadrangular	V-shaped	Multiple-banded	Total	p
	n=9	n=12	n=9	n=24	n=6	n=60	
LSTAI	1.40 ± 0.12^c,d^	1.38 ± 0.13^c,d^	1.19 ± 0.15^a,b,e^	1.19 ± 0.13^a,b,e^	1.45 ± 0.10^c,d^	1.29 ± 0.17	**p<0.001** **∗**
MSTAI	0,69 ± 0.13^c,d^	0.81 ± 0.17^c^	1.23 ± 0.23^a,b,d,e^	0.90 ± 0.13^a,c^	0.90 ± 0.09^c^	0.90 ± 0.22	**p<0.001** **∗**

LSTAI: lateral side thickness of acromial insertion; MSTAI: medial side thickness of acromial insertion.

Data are reported as mean ± SD. ANOVA; *α*: 0.05; independent t test; *∗*difference is statistically significant; post hoc; Tukey HSD test, Tamhane T2 test.

^a^ Difference with Y-shaped is statistically significant.

^b^ Difference with broad-banded is statistically significant.

^c^ Difference with quadrangular is statistically significant.

^d^ Difference with V-shaped is statistically significant.

^e^ Difference with multiple-banded is statistically significant.

**Table 3:** The mean ratios of LBL to MBL, CIW to AIW, and LSTAI to MSTAI according to CAL types

	Y-shaped	Broad-banded	Quadrangular	V-shaped	Multi-banded	Total	p
	n=9	n=12	n=9	n=24	n=6	n=60	
LBL/MBL	1.08 ± 0.13^b^	1.28 ± 0.11^a,c,d,e^	1.07 ± 0.07^b^	0.98 ± 0.12^b^	0.95 ± 0.08^b^	1.06 ± 0.16	**p<0.001** **∗**
CIW/AIW	2.53 ± 0.40^b^	1.42 ± 0.50^a,c,d^	2.59 ± 0.28^b^	2.92 ± 1.06^b^	2.80 ± 0.97	2.50 ± 0.96	**p<0.001** **∗**
LSTAI/ MSTAI	2.07 ± 0.27^b,c,d,e^	1.76 ± 0.28^a,c,d^	0.98 ± 0.13^a,b,d,e^	1.33 ± 0.16^a,b,c,e^	1.62 ± 0.18^a,c,d^	1.50 ± 0.40	**p<0.001** **∗**

Data are reported as mean ± SD. ANOVA; *α*: 0.05; independent t test; *∗*difference is statistically significant; post hoc; Tukey HSD test, Tamhane T2 test.

^a^ Difference with Y-shaped is statistically significant.

^b^ Difference with broad-banded is statistically significant.

^c^ Difference with quadrangular is statistically significant.

^d^ Difference with V-shaped is statistically significant.

^e^ Difference with multiple-banded is statistically significant.

**Table 4:** The comparison of CAL types in the current study with the types reported in the literature.

Study		Y-shaped	Broad-banded	Quadrangular	V-shaped	Multiple-banded
	N	%	%	%	%	%
Holt et al. [10]	48	42	8	48	-	2
Kopuz et al. [17]	110*∗*	44*∗∗*	29	27	-	** -**
Fagelman et al. [15]	7	28.5	28.5	43	-	** -**
Kesmezacar et al. [12]	80	41	23	14	11	11
Hu et al. [18]	9	11	44	44	-	-
Alraddadi et al. [13]	220	38	6	10	-	46
Current study	60	15	20	15	40	10

*∗*Kopuz et al. [17] studied CAL morphology in shoulders of neonatal cadavers whereas the other studies mentioned were executed in adult shoulders.

*∗∗* Kopuz et al. [17] designated two-banded CAL as “U-shaped ligament” in the relevant study.

## Data Availability

The data used to support the findings of this study are included within the article and are available from the corresponding author upon request.
